# Adverse clinical outcomes associated with double dose clopidogrel compared to the other antiplatelet regimens in patients with coronary artery disease: a systematic review and meta-analysis

**DOI:** 10.1186/s40360-018-0247-9

**Published:** 2018-09-03

**Authors:** Xiaojun Zhuo, Bi Zhuo, Shenyu Ouyang, Pei Niu, Mou Xiao

**Affiliations:** 1 Department of Cardiology, Affiliated Changsha Hospital of Hunan Normal University, The Fourth Hospital of Changsha, Changsha, 410006 Hunan People’s Republic of China; 2 Department of Pharmacology, People’s Hospital of Laibin, Laibin, 546100 Guangxi People’s Republic of China

**Keywords:** Double dose clopidogrel, Dual antiplatelet therapy, Ticagrelor, Prasugrel, Triple antiplatelet therapy, Coronary artery disease, Percutaneous coronary intervention

## Abstract

**Background:**

Recently, several newer antiplatelet treatment strategies have been used in patients with coronary artery disease (CAD). Apart from the dual antiplatelet therapy (DAPT) consisting of aspirin and clopidogrel, double dose clopidogrel (DDC), triple antiplatelet therapy (TAPT) consisting of aspirin, clopidogrel and cilostazol and other newer antiplatelet agents have shown to be effective in different ways. In this analysis, we aimed to systematically compare the adverse clinical outcomes and the bleeding events which were observed when DDC was compared to the other antiplatelet regimens in patients with CAD.

**Methods:**

English publications comparing DDC with other antiplatelet regimens were searched from MEDLARS/MEDLINE, EMBASE, www.ClinicalTrials.gov and Google Scholar. Adverse cardiovascular outcomes and bleeding events were the study endpoints. Statistical analysis was carried out by the RevMan 5.3 software whereby odds ratios (OR) with 95% confidence intervals (CIs) were calculated.

**Results:**

A total number of 23,065 participants were included. Results of this analysis showed major adverse cardiac events (MACEs), all-cause mortality, cardiac death, stroke, stent thrombosis, revascularization and myocardial infarction (MI) to have been similarly manifested in patients who were treated with DDC versus the control group with OR: 0.98, 95% CI: 0.78–1.22; *p* = 0.83, OR: 0.95, 95% CI: 0.77–1.17; *p* = 0.62, OR: 0.97, 95% CI: 0.79–1.20; *p* = 0.81, OR: 0.98, 95% CI: 0.65–1.48; *p* = 0.94, OR: 0.84, 95% CI: 0.40–1.75; *p* = 0.64, OR: 0.88, 95% CI: 0.52–1.49; *p* = 0.63, and OR: 0.89, 95% CI: 0.65–1.21; *p* = 0.45 respectively. Any minor and major bleedings were also similarly manifested.

When DDC was compared to DAPT, no significant difference was observed in any bleeding event with OR: 1.58, 95% CI: 0.86–2.91; *p* = 0.14. Even when DDC was compared with either ticagrelor or prasugrel or TAPT, still no significant difference was observed in terms of bleeding outcomes.

**Conclusions:**

In patients with CAD, adverse clinical outcomes were not significantly different when DDC was compared to the other antiplatelet regimens. In addition, bleeding events were also similarly manifested when DDC was compared to DAPT, TAPT or ticagrelor/prasugrel.

## Background

Coronary artery disease (CAD) is one among the most common non-communicable diseases affecting a large number of the elderly population around the globe [1]. As a measure of secondary prevention, several antiplatelet treatment strategies have been set up based upon the degree and type of intervention which was carried out. In patients with stable CAD where intervention was not required, a single antiplatelet agent was sufficient [2]. For those patients with acute coronary syndrome (ACS) or those patients undergoing percutaneous coronary intervention (PCI) with drug eluting stents (DES), dual antiplatelet therapy (DAPT) consisting of aspirin and clopidogrel has been the mainstay of treatment [3].

However, clopidogrel hyporesponsiveness [4] and platelet hyper-reactivity [5] have recently been observed in several subgroups of patients. Therefore, to overcome this problem, several new antiplatelet treatment strategies have been developed: Double dose clopidogrel (DDC) [6], triple antiplatelet therapy (TAPT) consisting of aspirin, clopidogrel and cilostazol [7] and other newer potential antiplatelet agents such as ticagrelor and prasugrel have been used [8].

Nevertheless, controversies have been observed with the use of DDC. Results of the CURRENT OASIS 7 Trial which was published in the New England Journal of Medicine showed no benefit of DDC in patients with ACS. However, a subgroup analysis of the same data (CURRENT OASIS 7) which was published in The Lancet indicated a beneficial effect of DDC in ACS patients following coronary stenting. However, DDC has never systematically been compared with the other antiplatelet agents.

In this analysis, we aimed to systematically compare the adverse clinical outcomes and the bleeding events which were observed with DDC versus the other antiplatelet regimens in patients with CAD.

## Methods

### Searched databases and searched strategies

MEDLARS or MEDLINE (Medical Literature Analysis and Retrieval System Online), EMBASE, www.ClinicalTrials.gov and Google Scholar were the online electronic databases which were searched for relevant English publications comparing DDC with other antiplatelet regimens in patients with CAD.

The following searched terms were used to retrieve publications:

- Double dose clopidogrel and coronary artery disease;
- Double dose clopidogrel and percutaneous coronary intervention;
- Double dose clopidogrel and acute coronary syndrome;
- Double dose clopidogrel and acute myocardial infarction;
- Double dose clopidogrel and dual antiplatelet therapy;
- Double dose clopidogrel and triple antiplatelet therapy;
- Double dose clopidogrel and cilostazol;
- Double dose clopidogrel and prasugrel;
- Double dose clopidogrel and ticagrelor;
- Double dose clopidogrel and antiplatelet agents.

The term ‘double dose’ was also replaced by the term ‘high dose’ in this search process.

### Inclusion and exclusion criteria

Studies were included in this analysis if:

- They compared double dose clopidogrel versus other antiplatelet agents in patients with CAD/PCI;
- They reported adverse clinical outcomes and bleeding events as their endpoints.

Studies were excluded from this analysis if:

- They were review articles, meta-analyses, case studies or letters to editors;
- They did not compare DDC with other antiplatelet agents;
- They did not report adverse clinical outcomes or bleeding events as their clinical endpoints; Instead, they only reported platelet activities;
- They were duplicated studies.

### Definitions, outcomes and follow-ups

DAPT: Dual antiplatelet therapy consisted of Aspirin and Clopidogrel;

TAPT: Triple antiplatelet therapy consisted of Aspirin, Clopidogrel and Cilostazol;

DDC: Double dose clopidogrel consisted twice the normal standard dose of clopidogrel given daily; that is, 150 mg clopidogrel.

The following outcomes were assessed:

- Major adverse cardiac events (MACEs) consisting of mortality, myocardial infarction (MI), repeated revascularization, or stroke;
- All-cause mortality;
- Cardiac death;
- MI;
- Stroke;
- Stent thrombosis;
- Revascularization (target vessel revascularization or target lesion revascularization);
- Any bleeding event consisting of any type of bleeding which was reported;
- Any minor bleeding consisting of any minor type of bleeding or minimal bleeding;
- Any major bleeding consisting of any type of major bleeding or serious bleeding.

The follow-up time period varied from study to study. The outcomes which were reported as well as the follow-up time periods have been listed in Table 1.

**Table 1:** Outcomes and follow-up periods

Studies	Outcomes reported	Follow-up periods	DDC versus control group
CURRENT OASIS 7 [11]	CV death, MI, or stroke (MACE); CV death, MI, stroke, total mortality, TIMI major bleeding, minor bleeding, fatal bleeding, intracranial bleeding	30 days	DDC versus SDAPT
ACCEL AMI [12]	Minor bleeding	30 days	DDC versus SDAPT
OPTIMUS2007 [13]	Bleeding complications	30 days	DDC versus SDAPT
CREATIVE [14]	MACE, all-cause death, cardiac death, MI, TVR, stroke, ST, major bleeding	18 months	DDC versus SDAPT
Chen2017 [15]	MACE, in-stent thrombosis, TVR, MI, cardiac death, bleeding events, mild bleeding, severe bleeding	12 months	DDC versus SDAPT
Khatri2013 [16]	Composite efficacy outcomes, any bleeding event	23 months	DDC versus prasugrel
OPTIMUS3 [17]	Major and minor TIMI defined bleeding, adverse drug events	7 days	DDC versus prasugrel
PRINCIPLE TIMI 44 [18]	TIMI major and minor bleeding, minor bleeding, hemorrhagic adverse events, MI	2 weeks	DDC versus prasugrel
Tailor2014 [19]	MACE, MI, ST, CV death, stroke	1 month and 571 days	DDC versus prasugrel
Chen2017 [15]	MACE, in-stent thrombosis, TVR, MI, cardiac death, bleeding events, mild bleeding, severe bleeding	12 months	DDC versus ticagrelor
Wu2017 [20]	MACE, minimal bleeding, minor bleeding	30 days	DDC versus ticagrelor
ACCEL AMI [12]	Minor bleeding	30 days	DDC versus TAPT
ACCEL DM [21]	Bleeding event	30 days	DDC versus TAPT
CREATIVE [14]	MACE, all-cause death, cardiac death, MI, TVR, stroke, ST, major bleeding	18 months	DDC versus TAPT
Ha2013 [22]	MACE	1 month	DDC versus TAPT
HOST ASSURE [23]	MACE, cardiac death, MI, stroke, ST, all-cause death, TLR, TVR, PLATO minor bleeding	30 days	DDC versus TAPT
Jeong2009 [24]	MACE, major and minor bleeding	30 days	DDC versus TAPT

Abbreviations: TAPT: triple antiplatelet therapy consisting of aspirin, clopidogrel and cilostazol, DDC: double dose clopidogrel, SDAPT: standard dual antiplatelet therapy, CV: cardiovascular, MI: myocardial infarction, ST: stent thrombosis, MACE: major adverse cardiac events, TIMI: thrombolysis in myocardial infarction, TVR: target vessel revascularization, TLR: target lesion revascularization

### Data extraction, quality assessment and statistical analysis

Following the search of publications by the PRISMA guideline [9], and after selection of the most suitable articles which were relevant to this analysis, data extraction was carried out by five independent reviewers. The following data were extracted:

The type of study, the number of participants assigned to the DDC group and the control group respectively, the time period when the participants were enrolled, the type of participants, the baseline features, the cardiovascular and bleeding outcomes which were reported, the total number of events in each subgroups, the follow-up time periods and the methodological quality of the trials.

Any disagreement which followed was resolved by consensus.

The methodological quality of the trials was assessed in accordance to the Cochrane Collaboration [10].

Statistical analysis was carried out by the RevMan 5.3 software whereby odds ratios (OR) with 95% confidence intervals (CIs) were calculated.

Heterogeneity was assessed by the (1) Q statistic test whereby a *p* value less than 0.05 was considered statistically significant and (2) the I^2^ statistic test whereby a low heterogeneity was denoted by a low I^2^ value and a high heterogeneity was represented by an increased value of I^2^.

Concerning the statistical models which were used, a fixed effects model was used if I^2^ was less than 50% whereas a random effects model was used if I^2^ was greater than 50%.

Sensitivity analysis was carried out by a method of exclusion whereby each trial was excluded one by one and a new analysis was carried out each time to be compared with the main results for any significant difference.

Since this analysis did not include a large volume of studies, publication bias was best assessed through funnel plots which were obtained from the RevMan software.

## Results

### Searched outcomes

After a careful search, a total number of 1514 publications were obtained. Following an assessment of the titles and abstracts, 1398 articles were eliminated since they were not related to this research topic.

One hundred and sixteen (116) full text articles were assessed for eligibility. Further elimination were carried out for the following reasons:

Meta-analysis (3), review of literature (5), case studies (3), letter to editors (4), did not compare DDC with other antiplatelet agents (32), did not report relevant adverse outcomes (25), and duplicated studies (30).

**Fig. 1:**
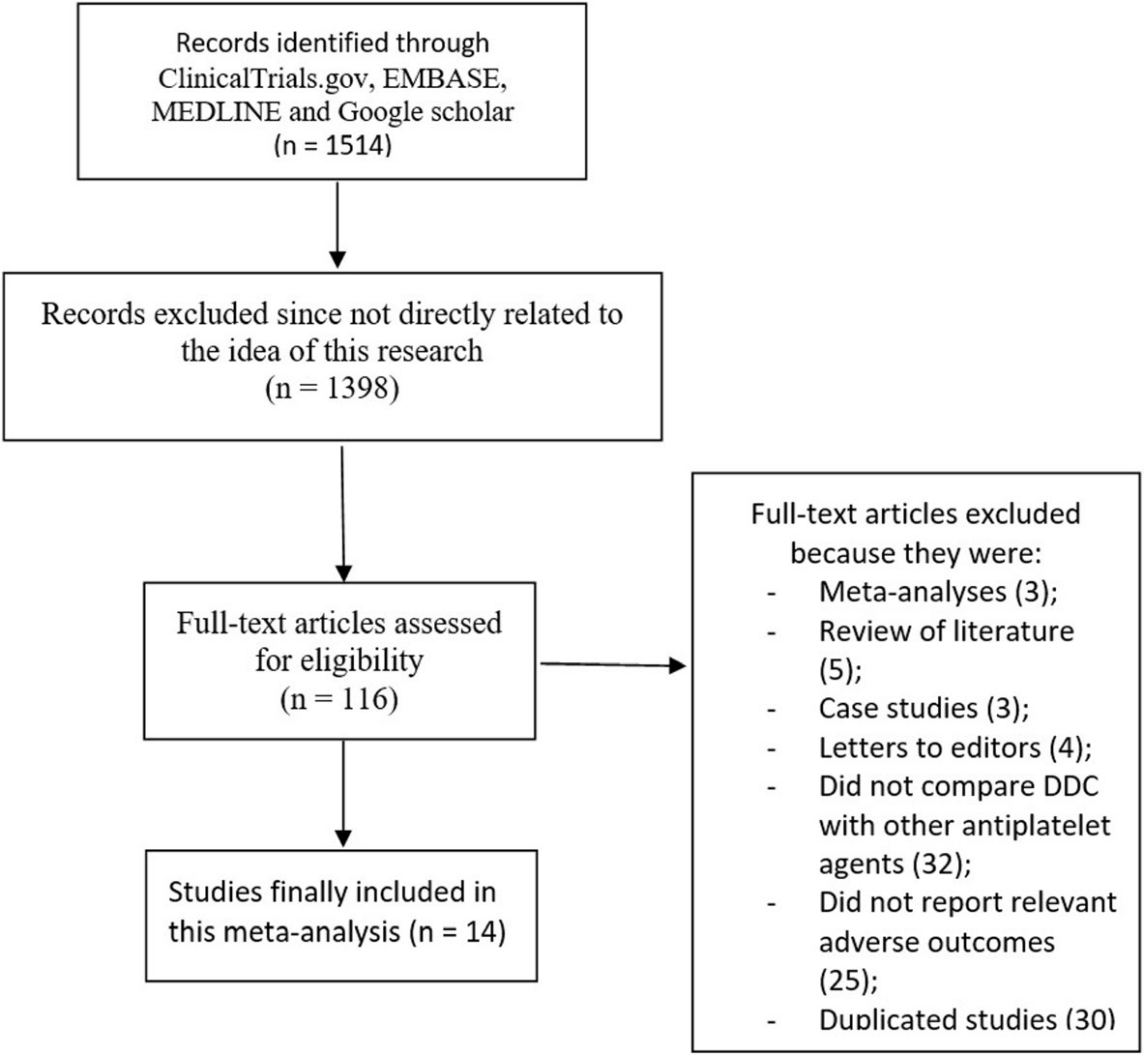
Flow diagram representing the study selection

Finally only 14 studies [11–24] were considered relevant for this analysis as shown in Fig. 1.

### General features of the studies

The general features have been listed in Table 2.

**Table 2:** General features of the studies

Studies	No of patients in the DDC group (n)	No of patients in control group (n)	Year of patients’ enrollment	Type of study	Type of participants
CURRENT OASIS 7	8560	8703	2006–2009	RCT	ACS + PCI
OPTIMUS2007	20	20	–	Pilot study	DM + CAD
Khatri2013	26	64	2009–2010	Retrospective study	CAD
OPTIMUS3	35	34	2008–2009	RCT	DM + CAD
PRINCIPLE TIMI 44	99	102	–	RCT	Any CAD with planned PCI
Tailor2014	52	54	2010–2012	RCT	CAD + ACS
Chen2017	50	57 + 46	2012–2014	OC	CAD
Wu2017	20	20	2014–2015	RCT	CAD with planned PCI
ACCEL AMI	30	30 + 30	–	RCT	AMI
ACCEL DM	39	41	–	RCT	DM + AMI undergoing PCI
CREATIVE	359	362 + 355	2012–2015	RCT	CAD + PCI
Ha2013	21	21	–	RCT	DM + PCI
HOST ASSURE	1876	1879	2010–2011	RCT	CAD + PCI
Jeong2009	30	30	–	RCT	CAD + PCI
Total number of patients (n)	11,217	11,848			

Abbreviations: ACS: acute coronary syndrome, PCI: percutaneous coronary intervention, DM: diabetes mellitus, CAD: coronary artery disease, AMI: acute myocardial infarction, RCT: randomized controlled trials, OC: observational studies; DDC: double dose clopidogrel

A total number of 23,065 participants were included in this analysis whereby 11,217 were assigned to the DDC group and 11,848 participants were assigned to the control group. In detail, the number of participants were assigned as followed: DDC (9019 participants) versus standard dual antiplatelet therapy (9172 participants), DDC (2355 participants) versus TAPT (2356 participants), and DDC (282 participants) versus ticagrelor/prasugrel (320 participants). Eleven studies were randomized trials whereas the other three studies were observational cohorts. The time period of patients’ enrollment varied from years 2006 to 2015 as shown in Table 2.

Patients with stable CAD, ACS, diabetes mellitus and those undergoing PCI were included in this analysis.

### Baseline features of the studies

The baseline characteristics have been reported in Table 3. Majority of the participants were of male gender with a mean age ranging from 58.1 to 64.9 years. Hypertension, dyslipidemia and diabetes mellitus among the patients varied from 26.0 to 100% as shown in Table 3. According to the baseline features, no significant difference was observed between the two groups of participants.

**Table 3:** Baseline features of the studies

Studies	Age (years)	Males (%)	HBP (%)	DS (%)	DM (%)	CS (%)
	DDC/C	DDC/C	DDC/C	DDC/C	DDC/C	DDC/C
CURRENT OASIS 7	61.2/61.2	76.0/74.9	59.4/58.8	40.3/40.3	22.3/22.2	37.5/36.6
OPTIMUS2007	64.0/59.0	60.0/70.0	90.0/95.0	90.0/95.0	100/100	15.0/20.0
Khatri2013	64.0/62.0	100/8.00	81.0/91.0	100/97.0	73.0/61.0	69.0/61.0
OPTIMUS3	61.3/61.3	68.6/68.6	94.3/94.3	94.3/94.3	100/100	20.0/20.0
PRINCIPLE TIMI 44	63.8/64.0	77.8/71.6	77.8/85.3	86.9/90.2	29.3/32.4	16.2/17.6
Tailor2014	63.0/63.0	82.7/74.1	82.7/74.1	88.5/83.3	34.6/29.6	67.3/77.8
Chen2017	59.8/60.8	62.0/59.6	56.0/56.1	26.0/28.1	30.0/31.6	38.0/35.1
Wu2017	62.7/60.4	70.0/75.0	70.0/50.0	60.0/80.0	30.0/45.0	30.0/55.0
ACCEL AMI	61.1/62.7	76.7/71.7	36.7/46.7	46.7/36.7	20.0/21.7	73.3/61.7
ACCEL DM	62.0/64.0	66.7/70.7	64.1/75.6	33.3/34.1	100/100	43.6/41.5
CREATIVE	58.1/58.5	61.0/59.3	61.0/65.8	68.5/64.5	32.0/33.8	38.2/36.5
Ha2013	62.3/64.9	71.4/67.7	76.1/71.4	42.5/33.3	100/100	23.8/23.8
HOST ASSURE	63.7/62.8	67.0/69.8	68.6/66.8	62.7/64.2	31.3/31.8	30.8/32.8
Jeong2009	63.0/63.0	66.7/66.7	50.0/53.3	20.0/20.0	16.7/30.0	60.0/36.7

Abbreviations: DDC: double dose clopidogrel, C: control group, HBP: high blood pressure, DS: dyslipidemia, DM: diabetes mellitus, CS: current smoker

### Adverse cardiovascular outcomes associated with double dose clopidogrel versus the control group

**Fig. 2:**
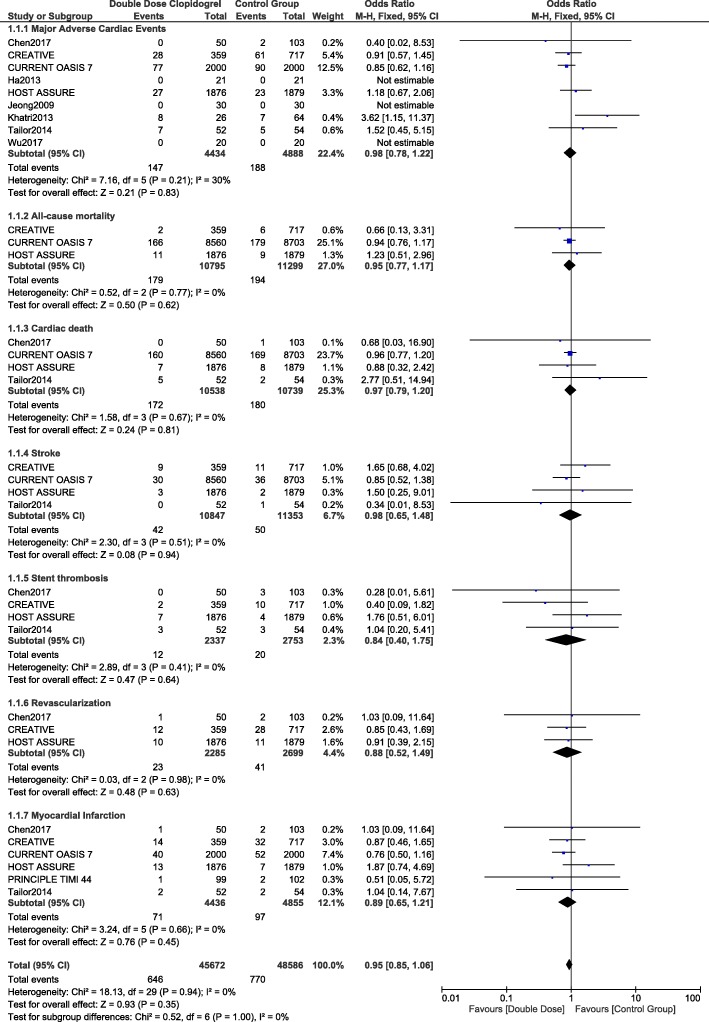
Comparing the adverse cardiovascular outcomes observed with double dose clopidogrel versus the other antiplatelet regimens

When the adverse cardiovascular outcomes were compared, MACEs, all-cause mortality, cardiac death, stroke, stent thrombosis, revascularization and MI were similarly manifested in patients who were treated with DDC versus the control group with OR: 0.98, 95% CI: 0.78–1.22; *p* = 0.83, OR: 0.95, 95% CI: 0.77–1.17; *p* = 0.62, OR: 0.97, 95% CI: 0.79–1.20; *p* = 0.81, OR: 0.98, 95% CI: 0.65–1.48; *p* = 0.94, OR: 0.84, 95% CI: 0.40–1.75; *p* = 0.64, OR: 0.88, 95% CI: 0.52–1.49; *p* = 0.63, and OR: 0.89, 95% CI: 0.65–1.21; *p* = 0.45 respectively as shown in Fig. 2.

### Bleeding associated with double dose clopidogrel versus the control group

**Fig. 3:**
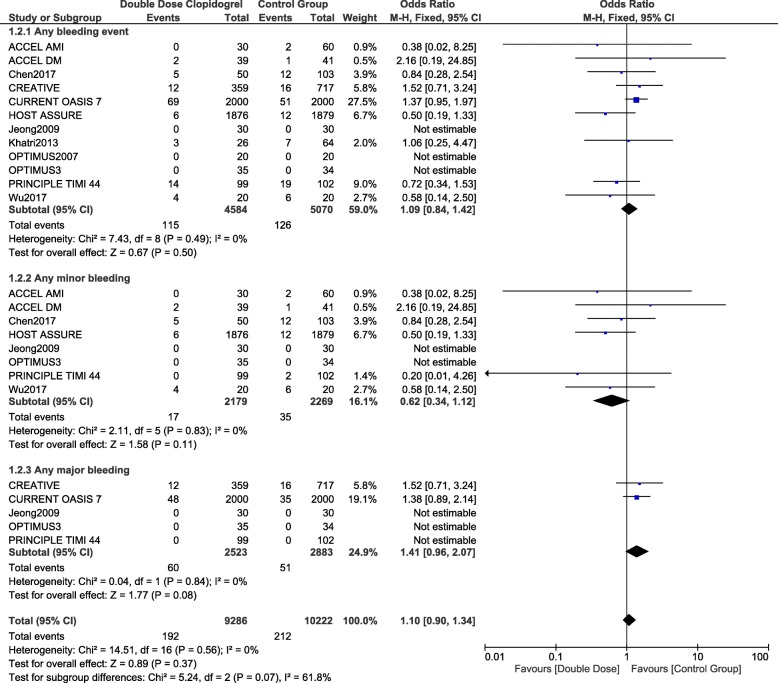
Comparing bleeding events observed with double dose clopidogrel versus the other antiplatelet regimens

The outcome ‘any bleeding event’ was not significantly different with DDC versus the control group with OR: 1.09, 95% CI: 0.84–1.42; *p* = 0.50. In addition, any minor bleeding and any major bleeding were also similarly manifested with OR: 0.62, 95% CI: 0.34–1.12; *p* = 0.11 and OR: 1.41, 95% CI: 0.96–2.07; *p* = 0.08 respectively as shown in Fig. 3.

Bleeding events were further analyzed whereby DDC was compared individually with different antiplatelet regimens.

### Bleeding associated with double dose clopidogrel versus standard dual anti-platelet therapy

**Fig. 4:**
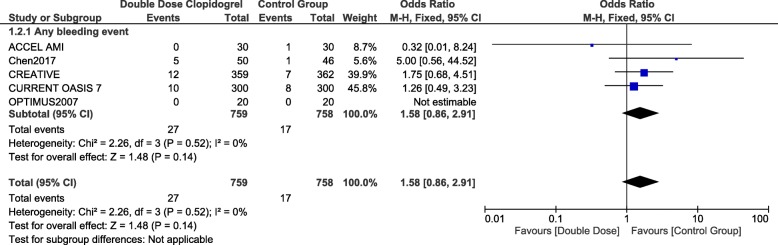
Comparing bleeding events observed with double dose clopidogrel versus the standard dual antiplatelet therapy

When DDC was compared with the standard dual antiplatelet therapy (Aspirin and clopidogrel), no significant difference was observed in any bleeding event with OR: 1.58, 95% CI: 0.86–2.91; *p* = 0.14 as shown in Fig. 4.

### Bleeding associated with double dose clopidogrel versus ticagrelor or prasugrel

**Fig. 5:**
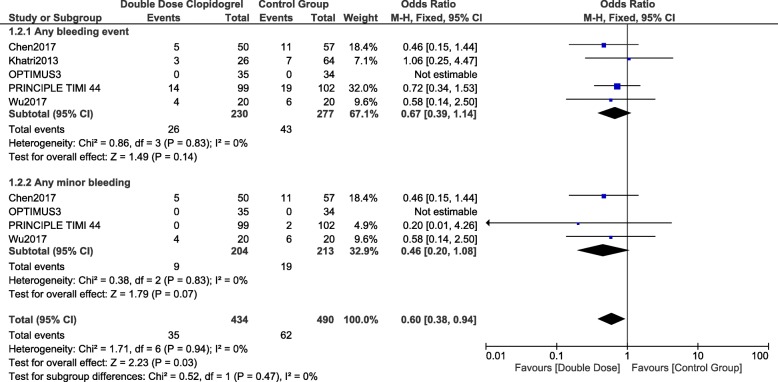
Comparing bleeding events observed with double dose clopidogrel versus ticagrelor or prasugrel

When DDC was compared with either ticagrelor or prasugrel, still no significant difference was observed in any bleeding events and any minor bleeding with OR: 0.67, 95% CI: 0.39–1.14; *p* = 0.14 and OR: 0.46, 95% CI: 0.20–1.08; *p* = 0.07 respectively as shown in Fig. 5.

### Bleeding associated with double dose clopidogrel versus triple therapy (aspirin, clopidogrel and cilostazol)

**Fig. 6:**
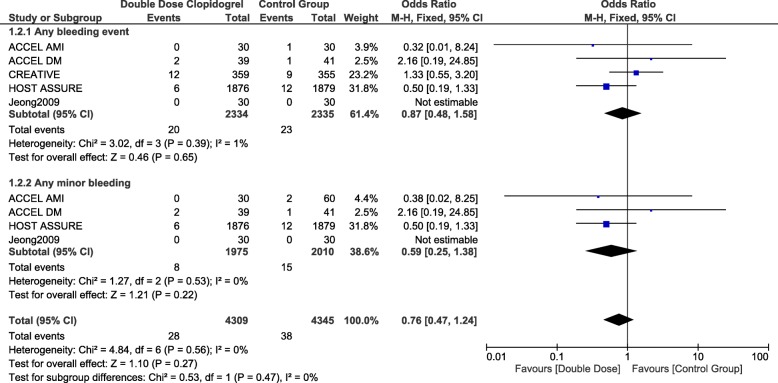
Comparing bleeding events observed with double dose clopidogrel versus triple antiplatelet regimen

When DDC was compared with TAPT, any bleeding event and any minor bleeding were not significantly different with OR: 0.87, 95% CI: 0.48–1.58; *p* = 0.65 and OR: 0.59, 95% CI: 0.25–1.38; *p* = 0.22 respectively as shown in Fig. 6.

**Fig. 7:**
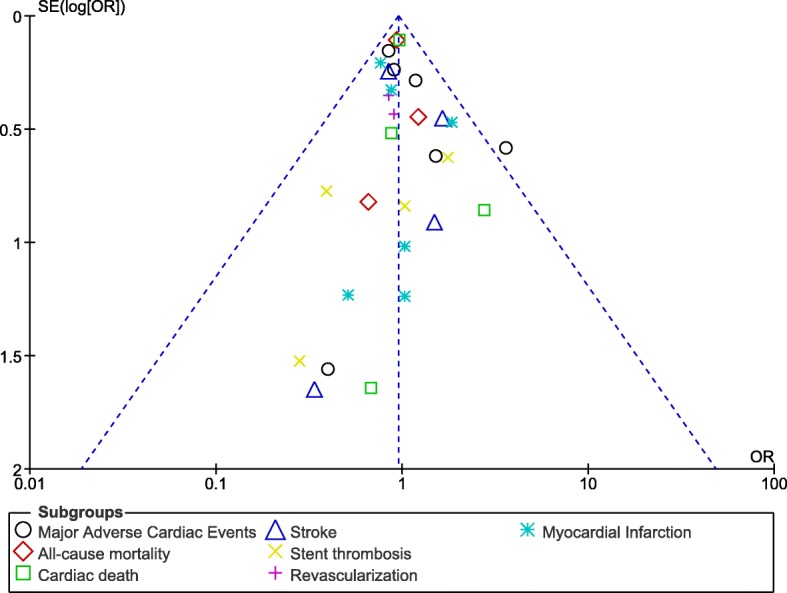
Funnel plot representing publication bias (A)

**Fig. 8:**
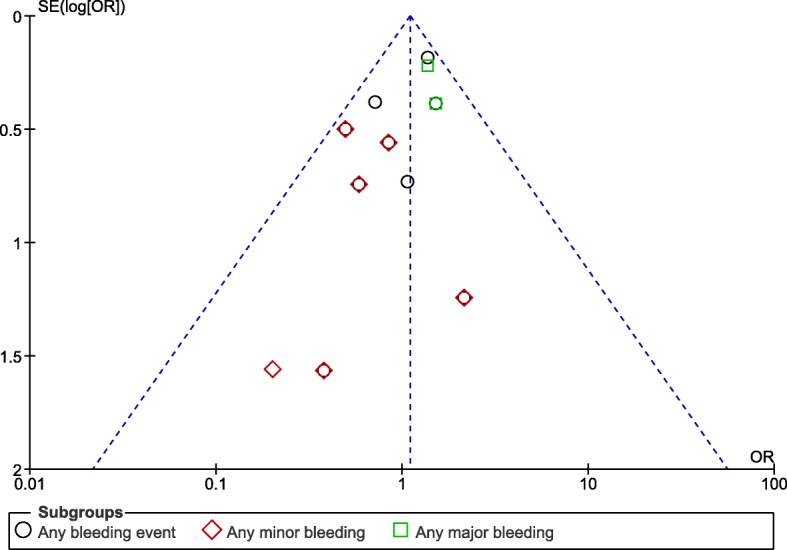
Funnel plot representing publication bias (B)

Sensitivity analyzes showed consistent results accordingly. A low evidence of publication bias was observed which was visually assessed through funnel plots (Figs. 7 and 8) which were directly obtained through RevMan.

## Discussion

This is the first analysis to systematically compare DDC versus the other antiplatelet agents. The current results showed that adverse clinical outcomes were not significantly different with DDC versus the other antiplatelet regimens. In addition, bleeding events (including major and minor bleeding) were also similarly manifested.

Results of the Adjunctive Cilostazol Versus High Maintenance Dose Clopidogrel in Patients With AMI (ACCEL-AMI) Study showed that TAPT demonstrated greater platelet inhibition compared to DDC [12]. However, no major cardiovascular or bleeding outcomes were observed in any of the groups supporting the results of this current analysis. The Adjunctive Cilostazol versus double-dose ClopidogrEL in Diabetes Mellitus study (ACCEL-DM) also showed that when cilostazol was added to DAPT, the new regimen showed greater platelet inhibition in comparison to DDC [21]. However, no major bleeding was observed in any of the two groups.

Similarly, in the Gauging Responsiveness with A VerifyNow assay-Impact on Thrombosis And Safety (GRAVITAS) randomized study [25], whereby a total number of 2214 patients were assigned to DDC and standard clopidogrel dose, the former did not decrease the incidence of adverse cardiac outcomes in those patients who underwent PCI. Severe or moderate bleeding were also not significantly different.

Results of the Clopidogrel Response Evaluation and AnTi-platelet InterVEntion in High Thrombotic Risk PCI Patients (CREATIVE) Trial which compared DDC with that of the standard DAPT or cilostazol associated TAPT showed the latter to significantly improved outcomes [14]. However, no significant difference was observed when DDC was compared to the standard DAPT.

In contrast, in the CURRENT OASIS 7 randomized trial, where DDC (whereby 8560 patients were assigned to) was compared with the standard DAPT (where 8703 patients were assigned to) in patients with acute coronary syndrome, the former significantly improved cardiovascular outcomes and stent thrombosis [11]. In addition, major bleeding was significantly higher with DDC during a follow-up time period of 30 days. However, it should be noted that in the CURRENT OASIS 7, the patients were also exposed to a low versus a high dosage of aspirin in addition to the DDC. However, in this current analysis, most of the studies included patients who did not receive a high dosage of aspirin.

Nevertheless, other studies have shown an impact of the CYP2C19 variant to also have interacted with platelet reactivity. For example, the Accelerated Platelet Inhibition by a Double Dose of Clopidogrel According to Gene Polymorphism study showed that among post-PCI treated patients who received DDC, carriage of CYP2C19 variant was associated with a high platelet reactivity which might have shown that DDC was non-inferior to the standard DAPT or TAPT [26]. However, the RESET GENE Trial showed this high treatment platelet reactivity to be completely abolished by prasugrel [27].

In addition, the Atorvastatin and Clopidogrel High Dose in stable patients with residual high platelet activity (ACHIDO) study showed that a high dose of atorvastatin significantly improved the pharmacodynamics effect of DDC [28], which was ignored in this current study.

### Novelty

This analysis is new because it is the first research paper to systematically compare DDC with the other antiplatelet regimens in patients with coronary artery disease. In addition, this is an important piece of information which might contribute to the literature of cardiovascular diseases. DDC was compared with the standard dual antiplatelet regimen, the triple antiplatelet regimen, and newer antiplatelet drugs such as prasugrel and ticagrelor which might represent a new feature. Moreover, a low level of heterogeneity was reported among almost all the subgroups, which might further contribute to the novelty of this analysis.

### Limitations

Limitations were as followed: Several trials consisted of a very small number of participants which might have been affected by larger trials. However, the proportion of participants were adjusted in larger trials to compensate for the small number of participants in other studies in order to have a final fair result. Different studies had different follow up time periods, and this might have influenced the final outcomes following statistical analysis. In addition, a few of the original studies were pilot studies whereby crossing over of clopidogrel and the control group was reported. This could have influenced the results to a minor extent. Moreover, major and minor bleedings were not reported when a few antiplatelet agents were compared with DDC since data concerning major and minor bleeding were missing in the original papers or they were reported in only one study and a comparison would have not been possible. At last, patients with different stages of coronary disease or intervention were combined and assessed: following PCI, patients with stable coronary artery disease, patients with AMI and other ACS were all together systematically analyzed.

## Conclusions

In patients with CAD, adverse clinical outcomes were not significantly different when DDC was compared to the other antiplatelet regimens. In addition, bleeding events were also similarly manifested when DDC was compared to DAPT, TAPT or ticagrelor/prasugrel. Larger upcoming trials should be able to confirm this hypothesis.

## Abbreviations

CAD: Coronary artery disease
DAPT: Dual antiplatelet therapy
DDC: Double dose clopidogrel
PCI: Percutaneous coronary intervention
TAPT: Triple antiplatelet therapy
